# Longitudinal Patterns of Adherence to Oral HIV Pre-Exposure Prophylaxis Among At-Risk Women in Tanga, Tanzania: A Convergent Analysis of Influencing Factors and Optimization Strategies

**DOI:** 10.1007/s10461-026-05187-2

**Published:** 2026-06-05

**Authors:** Wigilya P. Mikomangwa, Emmy Metta, Kåre Moen, Elia J. Mmbaga, Melkizedeck Thomas Leshabari, Stephen Matthew Kibusi, Christopher H. Mbotwa, Christopher R. Sudfeld, Muhammad Bakari, Appolinary A. R. Kamuhabwa, Gideon Kwesigabo

**Affiliations:** 1 Department of Epidemiology and Biostatistics, Muhimbili University of Health and Allied Sciences, Dar es Salaam, Tanzania; 2 Department of Community Medicine and Global Health, University of Oslo, Oslo, Norway; 3 Department of Behavioural Sciences, Muhimbili University of Health and Allied Sciences, Dar es Salaam, Tanzania; 4 Department of Public Health, University of Dodoma, Dodoma, Tanzania; 5 Mbeya College of Health and Allied Sciences, University of Dar es Salaam, Mbeya, Tanzania; 6 Department of Global Health and Population, Harvard T. H. Chan School of Public Health, Boston, USA; 7 Department of Internal Medicine, Muhimbili University of Health and Allied Sciences, Dar es Salaam, Tanzania; 8 Department of Clinical Pharmacy and Pharmacology, Muhimbili University of Health and Allied Sciences, Dar es Salaam, Tanzania

**Keywords:** Pre-exposure prophylaxis, HIV, Female sex workers, Adherence, Optimizing strategies

## Abstract

There is scarcity of evidence on the changes over time of adherence to oral HIV pre-exposure prophylaxis (PrEP) among female sex workers. Therefore, we assessed the changes of adherence to PrEP over 12 months and influencing factors for suboptimal adherence and explored strategies to optimize adherence to oral PrEP among female sex workers in Tanzania. Convergence analysis was conducted involving 313 respondent driven sampled participants in the city of Tanga. Adherence was assessed at months 1, 6, and 12. In month 12 we conducted 26 in-depth interviews in parallel with quantitative interviews. A modified Poisson regression and thematic analysis was conducted for quantitative and qualitative data respectively. The prevalence of suboptimal adherence at month 1 was 19.5% (95% CI 14.1–26.3), with a significant increase to 91.8% (95% CI 87–94.9) at month 6 and 94.7% (95% CI 90.3–97.1) at month 12. Sub-optimal adherence was influenced by: Medical and medication related barriers, mobility and travel, limited access and availability of PrEP pills, lifestyle-related factors, inadequate PrEP knowledge and low perceived benefits of PrEP, non-disclosure PrEP use and, low perceived HIV risk. Participants recommended modifying PrEP dosing, introducing multiple dosing options at the PrEP care clinic, enhancing counselling sessions, and ensuring continuous availability of PrEP pills. The prevalence of suboptimal PrEP adherence is high among female sex workers in Tanzania. The study underscores the need for implementation research on the introduction of multiple dosing modalities, coupled with improved availability and accessible PrEP services and tailored counselling programs.

## Background

The United Nations’ global targets aim to end AIDS as a public health threat by 2030. It strives to reduce new HIV infections to fewer than 370,000, with 95% of people at substantial risk of HIV, including female sex workers, having access to and using appropriate, prioritised, person-centred, and effective combination prevention options by 2025 [1]. Currently, only 50% of sex workers worldwide are covered and use at least two preventive services, along with pre-exposure prophylaxis (PrEP) [1], and most use them sub-optimally [2]. Despite the availability of various HIV prevention options such as daily oral PrEP, condoms, and HIV self-screening, the global targets remain unachieved, with new HIV infections still high at 1.3million in 2024, with a prevalence of 2.7% among sex workers [3].

Effective PrEP use through maintaining sufficient drug levels to provide maximum protection from HIV infection during times of high risk [4], is fundamental to reaching the global goal of reducing new HIV cases by 90% [1]. Notably, the protective effect of daily oral PrEP heavily relies on adherence [5]; women need to take 6–7 PrEP doses per week to be considered adherent and adequately protected [6]. However, adhering to daily PrEP can be challenging, which lessens its effectiveness both for individuals and populations [5, 7]. Globally, suboptimal PrEP adherence is most common in the Asia -Pacific region (53.2%), followed by sub-Saharan Africa (51.7%). Female sex workers exhibit the highest rates of suboptimal adherence among key populations, accounting for 62.4%. Emerging evidence shows that PrEP adherence drops significantly within the first six months of initiation [8, 9]. Factors such as socioeconomic barriers, complex dosing routines, concerns about side effects, and structural and behavioural issues contribute to suboptimal PrEP adherence among female sex workers [10, 11].

Adherence to daily oral PrEP is a complex process that includes uptake (PrEP access and initiation), executed adherence (day-to-day use of the PrEP pill), and persistence (sustained PrEP pill use over time), each of which requires its own optimization strategy [5, 12]. A recent network meta-analysis recommended a multi-component intervention for improving PrEP adherence, such as HIV self-testing with adherence feedback elements [13]. However, in that study, female sex workers were underrepresented. Only 1 of the 21 studies analysed included female sex workers, indicating the need to develop strategies to improve PrEP adherence by involving this group. Additionally, expanding the variety of PrEP dosing modalities, such as the dapivirine vaginal ring and long-acting injectable PrEP, is expected to address suboptimal adherence [14]. Despite the availability of additional preventive options for people at substantial risk of HIV globally, daily oral PrEP remains the primary biomedical prevention strategy in most sub-Saharan African countries, including Tanzania [6]. However, switching from daily oral PrEP requires maintaining 7-day adherence and a 2-day adherence period (after injection of cabotegravir and lenacapavir, respectively) to provide protection [6, 14]. Therefore, monitoring adherence over time is essential to maximize its impact in reducing overall population-level HIV incidence [7]. Currently, there is limited published data in sub-Saharan Africa, including Tanzania on the longitudinal pattern of PrEP adherence among at risk-women including female sex workers.

Understanding changes in PrEP adherence over time, as well as the influencing factors is crucial in designing client-centered interventions, especially for female sex workers who do not prefer injections, lack access or cannot afford them [4]. Therefore, we assessed the changes in adherence over 12 months, influencing factors and explored strategies to improve adherence to oral PrEP among female sex workers in Tanzania.

## Methods

### Study Design and Setting

We analyzed longitudinal data collected from the quasi-experimental trial for PrEP roll-out in Tanzania (PREPTA), which involved female sex workers and men who have sex with men in the cities of Dar es Salaam (intervention) and Tanga (control). The PREPTA project was implemented between March 2021 and June 2023 and aimed to assess the effectiveness of a mobile health application for improving daily oral PrEP adherence and retention in PrEP services [15]. To understand the real-life PrEP care and PrEP use without the influence of intervention in Tanzania since its roll-out in 2021, we analysed adherence levels and their determinants among female sex workers in the control group (city of Tanga) who returned for scheduled follow-up visits. Also, due to the differences in the sexual behaviour and HIV risks between female sex workers and men who have sex with men. We mainly focused our analysis on the former than the later to understand what triggers them to use or not use PrEP as per the prescription. We also considered that women are at higher risk of suboptimal adherence than other members of key population [8, 9].

**Fig. 1:**
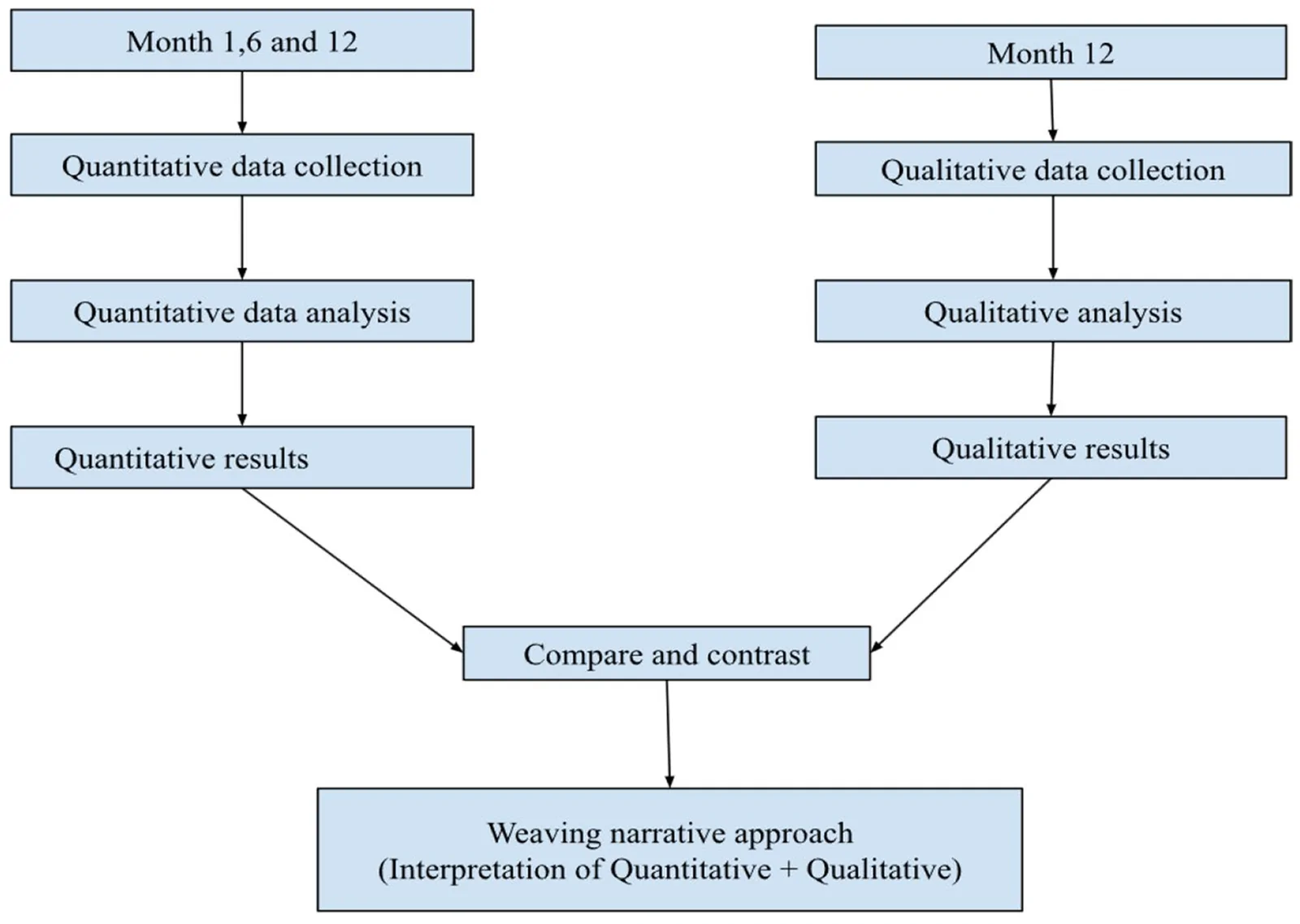
Convergence model

Participants in Tanga were recruited between February and April 2022 and were followed for up to 12 months [2]. The study was conducted at one of the clinics, which was centrally located in the city and was easily accessible. The enrolled participants were invited for scheduled interviews at the health center during follow-up months 1, 6 and 12. In month 12 of follow-up, we conducted in-depth interviews (IDIs) to explore the PrEP use experience, perspectives and opinions shaping adherence and strategies to improve adherence to daily oral PrEP among female sex workers. The qualitative interviews (IDI) were conducted in parallel with the quantitative interviews at the month 12. The qualitative and quantitative data were analyzed separately, and the results were converged and interpreted on the theme-by-theme basis as described in Fig. 1.

### Study Population, Sample Size and Sampling Techniques

The study involved women who were residents of Tanga and who reported having been engaged in sex work for at least 3 months preceding the study. A total of 313 HIV negative female sex workers aged 18 years or older who met the criteria for PrEP use as described in the guideline for HIV management [16] were recruited using respondent-driven sampling as described in our previous publications [2, 15]. The IDI involved participants who were purposefully sampled from the control group of cohorts of female sex workers. The sample size for the IDIs was determined by information saturation. Overall, 26 female sex workers participated in the IDIs.

### Quantitative Data Collection

Quantitative data were collected using an electronic questionnaire at baseline (month 0), and at months 1, 6 and 12. At baseline, we collected data on the socio-demographic, structural and sexual behaviour characteristics. During follow-up visits at months 1, 6 and 12, data were collected on PrEP use, adherence and reasons for missing PrEP doses. Adherence was assessed through a self-rating method, where participants estimated the percentage of time they believed they could take the PrEP pills as required. This self-rating adherence is a form of self-report measure that uses a single item to measure adherence. Participants were asked to consider a specified period and estimate on a continuum, the percentage (0% to 100%) of medication doses they had taken as prescribed [17]. Thus, participants were asked to rate the percentage of time they used the pills as prescribed. The following item was used: “*Thinking about the past 4 weeks, what percentage of the time were you able to take all of your PrEP medications as prescribed? (0% to 100 per cent*)”. This adherence measure is equivalent to the visual analogy scale, which is easy to administer and correlates with electronic medication monitoring, unannounced pill counts and short recall periods (3 to 4 days) [17, 18]. Additionally, we used self-report recall of missing doses over 3-day and 30-day periods to assess adherence as described previously [2].

Participants who reported missing any PrEP dose were asked to provide the reason(s) for skipping the pills. The reasons for missing PrEP doses were collected as multiple response options using a 4-point Likert scale ranging from “never”, “rarely”, “sometimes”, and “often”, i.e., participants rated the reason (s) for missing doses among the given choices. These choices were based on published literature on reasons for poor adherence in clinical trials and demonstration projects [10].

### Qualitative Data Collection

The IDIs were conducted at the 12-month follow-up, using Swahili, Tanzania’s national language. To ensure privacy, confidentiality and quality of voice recordings, interviews took place in a dedicated room at the health center and were recorded with a Dictaphone application [2] each lasting between 15 and 40 min. To avoid interviewer bias and to gather in-depth opinions on strategies for improving adherence, we maintained consistency in asking questions through probes, open-ended queries while avoiding overexplaining. We also documented pre-conceptions and prior knowledge in a field journal. To gather diverse and rich data on circumstances that influenced adherence and strategies to optimize adherence, we recruited female sex workers with different adherence statuses and PrEP use histories (continuing or stopped). Before each interview, the interviewer assessed adherence via self-report by asking whether the participant had missed any PrEP dose in the past three days. Participants were considered non-adherent if they missed any dose, and adherent if not. Their age and education level were also recorded. We sampled those who reported having stopped PrEP to explore their views on strategies to improve adherence if they choose to re-initiate PrEP use.

### Study Variables

#### Exposure Variables

The independent variables consisted of sociodemographic characteristics, PrEP self-efficacy, PrEP stigma, and social support. The sociodemographic characteristics of participants included age, education level, marital status, and whether they had children. PrEP self-efficacy was measured with six items, each rated on a 5-point scale from 0 (“not at all confident”) to 4 (“very confident”), with a reliability of 0.84. PrEP stigma was evaluated using a 10-item instrument with responses on a 5-point Likert scale (1 = “strongly disagree” to 5 = “strongly agree”), with the internal consistency of the scale being Cronbach’s α = 0.88; scores below 30 were categorized as low stigma. Social support was assessed with an adapted version of the Duke-UNC Functional Social Support Questionnaire (FSSQ), consisting of 8 items scored on a 5-point Likert scale (1 = “much less than I would like” to 5 = “as much as I like”). The measure showed good reliability (Cronbach’s α = 0.88); a total score below 32 was considered to reflect adequate social support [2].

#### Study Outcome

The primary outcome was changes in adherence to PrEP, as assessed at months 1, 6, and 12 after initiation. Sub-optimal adherence was defined as a self-rated PrEP use as < 80% or if one missed any dose in the previous 3 days or missed more than 6 doses of PrEP in the last 30 days (equivalent to < 80% pills per month) [2]. This definition aligns with the recommendation of 6–7 pills weekly over 4 weeks for optimal protection against HIV among female sex workers [19]. Also, the definition is consistent with the one employed in other studies in East Africa, where they used a cut-off of 80% to define optimal adherence to PrEP [11]. Reasons for suboptimal adherence were considered if the participant responded “rarely”, “sometimes” or “often”. We examined factors associated with long-term suboptimal adherence to PrEP, defined as suboptimal adherence at months 6 and 12 post-PrEP initiation. These time points were selected because, by month 6, participants are more likely to have established a routine, managed any side effects, experienced early barriers (such as side effects, pill fatigue, stigma, uncertainty about the need to use PrEP) and integrated PrEP into their daily lives, unlike at month 1 (which only indicates initial behavioural adjustment) [20]. Additionally, by month 6, participants would typically have attended at least two clinic visits for PrEP refills [6].

#### Quantitative Data Analysis

The descriptive analysis of the socio-demographic characteristics and reasons attributed to missing PrEP doses was presented as frequencies and proportions, while the median with interquartile range was used for continuous variables. We used self-rated adherence to identify factors associated with suboptimal adherence at months 6 and 12. A modified Poisson regression model with robust standard errors was employed to identify factors associated with sub-optimal adherence to PrEP. Variables with a p-value ≤ 0.25 in bivariate analysis were included in the multivariable model. Reasons were deemed common if the proportion was 10% or higher. All analyses were conducted using STATA version 18.

### Qualitative Data Analysis

The recorded IDIs were retrieved from the services for sensitive data (TSD) by the lead author, transcribed verbatim, and checked for completeness before analysis. The transcribed data were manually analysed thematically following Braun and Clarke’s approach to reflexive thematic analysis [21]. The transcripts were read and re-read to become familiar with the content of the dataset. The initial codebook was generated inductively by the lead author and reviewed. Every piece of relevant data was coded during the initial coding (open coding). The initial codes were then reviewed by re-reading the transcripts. Codes capturing lived experiences, perspectives and opinions were used to identify the reasons influencing adherence patterns to PrEP. The codebook was reviewed and enriched by a second person, while a third person reviewed the generated findings. The analysis was an iterative process. We grouped the codes that had similar patterns and overlaps to create sub-themes. Then, we reviewed potential themes by comparing them with the refined codes. Irrelevant codes were discarded or reallocated to another theme. Themes were refined and labelled deductively i.e., the obtained themes were labelled and interpreted in the light of the Health Belief Model (HBM) and supported by relevant quotes which were translated from Swahili to English. Moreover, themes related to recommendations on the strategies for optimizing PrEP adherence were labelled inductively i.e. labelled directly from the data itself without alignment to any pre-existing theory or framework or model. The lead author drafted the initial report, and the coauthors reviewed it. The findings are presented following the identified themes, and direct quotations are used to provide clarifications from the participants’ own views.

### Convergence Analysis

The triangulation of the quantitative and qualitative findings was conducted using a convergence model as described in Fig. 1. The quantitative results and qualitative findings were integrated to generate integrated insights i.e. meta-themes that explained the circumstances and perspectives shaping adherence to PrEP among female sex workers. The metathemes were generated by following the thread where the developed themes were followed across the quantitative insights (findings). We then assessed whether the components of quantitative data and themes converge (agree), expand, offer complementary information or dissonance. We also integrated and interpreted the quantitative results and qualitative themes by narrative approach: to allow writing of both quantitative and qualitative findings on the theme-by-theme basis or concept-by-concept basis we used the weaving narrative approach. To summarize the findings of the convergence we used a simplified joint display table which included the quantitative results, qualitative themes and meta-themes.

## Results

### Participants’ Characteristics

A total of 313 female sex workers were enrolled in the study with a baseline mean (SD) age of 27.7 (6.2) years. During the follow-up period, 164 attended the interview at month 1, 195 at month 6, and 188 at month 12. Most participants had never married (222/313, 70.8%), lived with family (237/313, 76.0%), and relied on sex work as their only source of income (163/313, 52.1%). The majority (254/313, 81.4%) perceived themselves to be at medium to high risk of HIV, and 76.5% (241/313) reported low PrEP stigma. About a quarter (80/313, 25.6%) of participants reported having been arrested by the police. At baseline, more than half (167/313, 53.7%) had travelled to other cities for sex work over the past 6 months. The participant characteristics at months 0,1, 6 and 12 did not differ significantly Table 1.

### Characteristics of Participants Who Took Part in In-depth Interviews

Table 2 describes the characteristics of IDI participants. The IDI involved 26 female sex workers with a mean age (SD) of 30.6(6.6) years. Among participants, 14 reported having suboptimal adherence, three (3) were adherent and nine (9) discontinued PrEP use. Participants who were either non-adherent or adherent reported continuing to use PrEP. The highest level of education attained was tertiary education.

### Longitudinal Patterns of Suboptimal Adherence Among Female Sex Workers

**Fig. 2:**
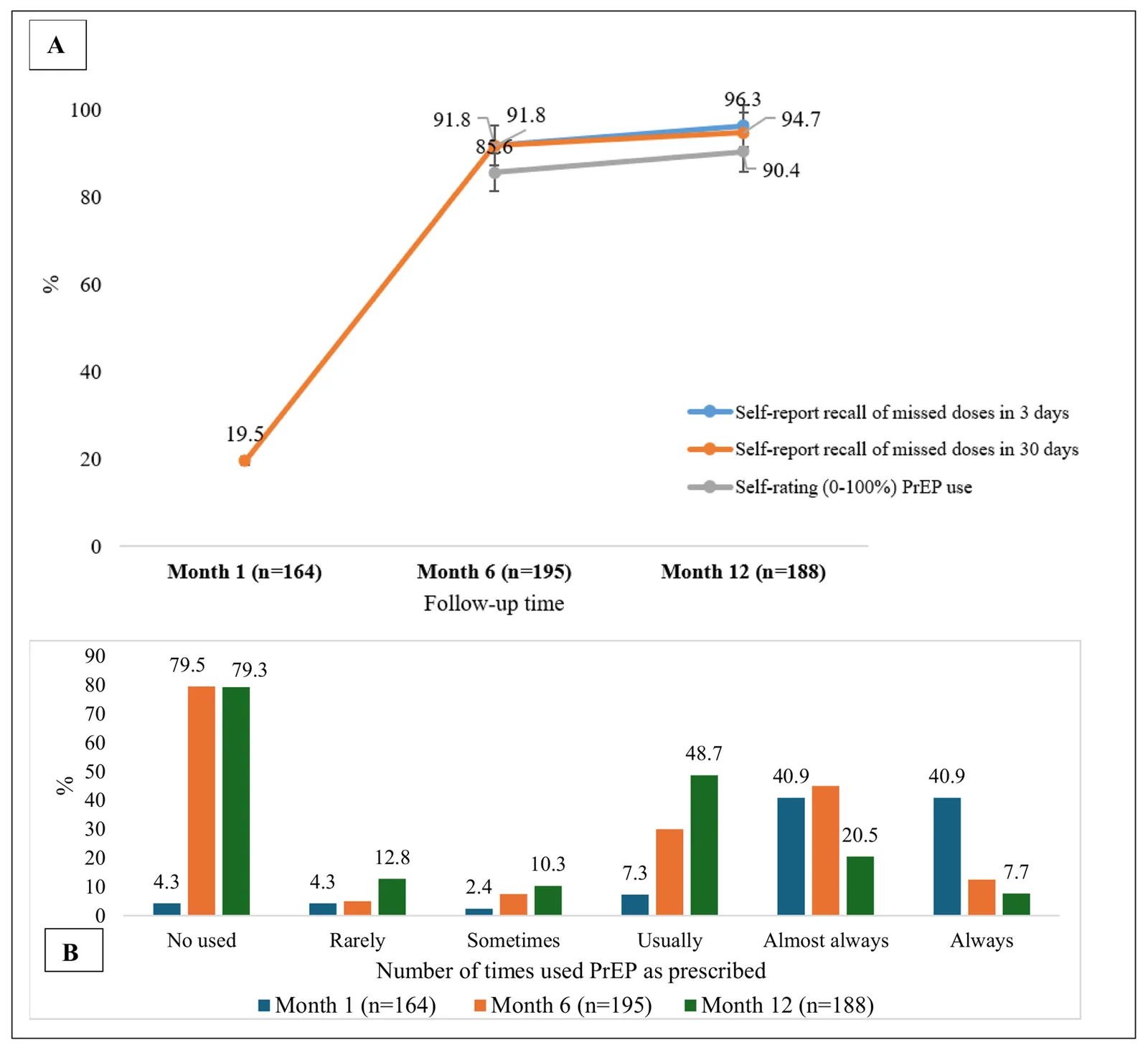
**A** Prevalence of suboptimal adherence to PrEP among female sex workers at three different points in time (months 1, 6 and 12) measured through three different types of self-reported adherence. **B** Proportion of the number of times female sex workers used PrEP doses as prescribed, as reported at three time points of month 1, 6 and 12

The prevalence of suboptimal adherence at month 1, as measured by self-reported recall of missed doses in the previous 30 days, was 19.5% (95% CI 14.1–26.3), with a significant increase to 91.8% (95% CI 87–94.9) at month 6 and 94.7% (95% CI 90.3–97.1) at month 12. When measured using self-reported recall of missed doses in the previous 3 days, the prevalence of suboptimal adherence at month 6 was 91.8% (95% CI 87–94.9), with a non-significant increase to 96.3% (95% CI 92.3–98.2) at month 12. Using a self-rated scale, the prevalence of suboptimal adherence at month 6 was 85.6% (95% CI 80.0–89.9) and increased to 90.4% (95% CI 85.2–93.9) at month 12, as shown in Fig. 2A. The 95%CI for the prevalence of suboptimal adherence between months 6 and 12 overlapped, indicating that they were not statistically significant.

Most participants did not use any PrEP dose in the last 30 days; the proportions being 79.5% (155/195) and 79.3% (149/188) at months 6 and 12, respectively. Of those who self-reported having used PrEP (at month 6, n = 40 and at month 12, n = 39), 5 (12.5%) and 3 (7.7%) reported always using PrEP as prescribed at months 6 and 12, respectively, as described in Fig. 2B.

**Fig. 3:**
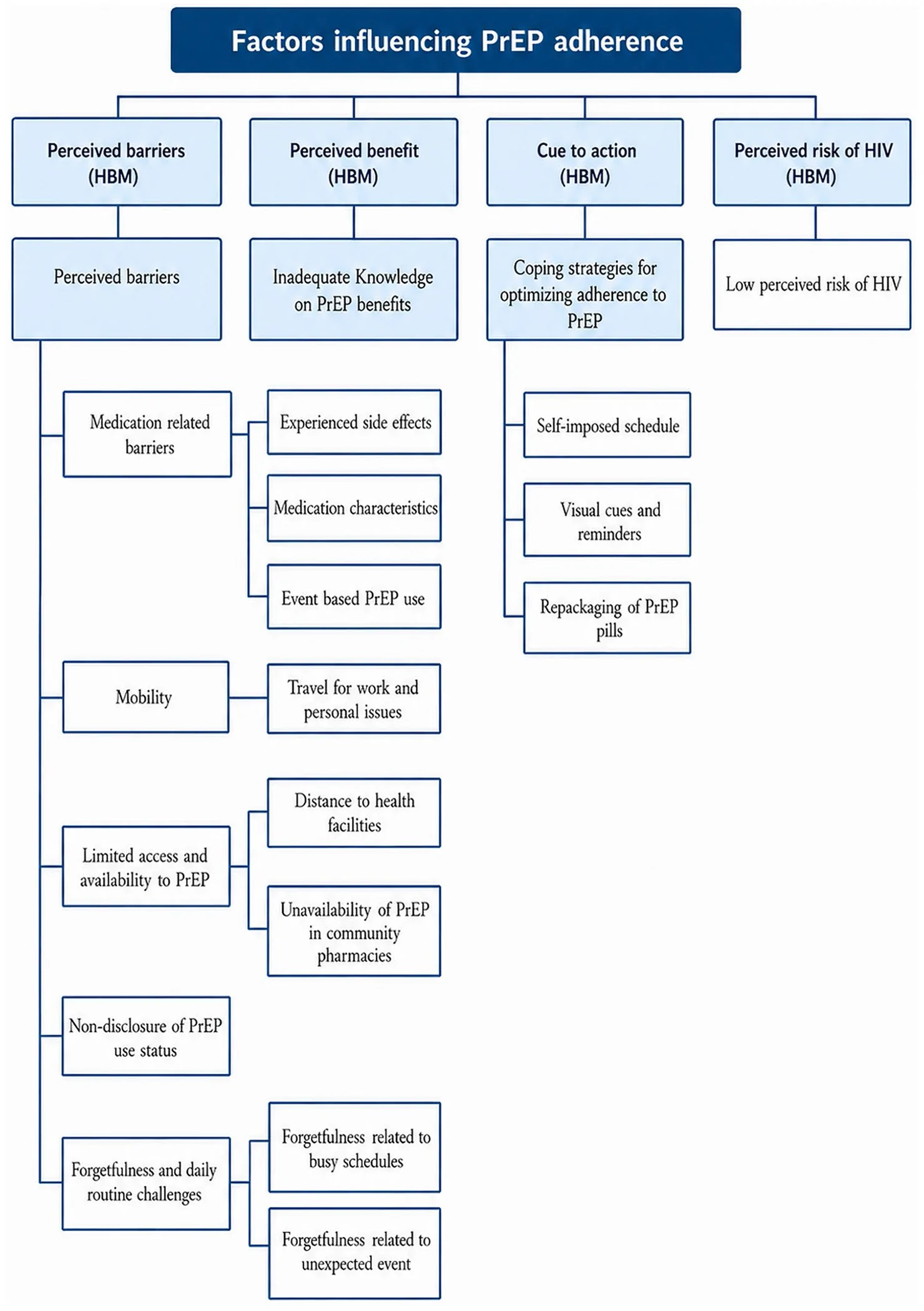
Distribution of themes and sub-themes on the factors influencing adherence to PrEP by corresponding theoretical construct in the health belief model

We then separately analyzed qualitative data. Eight themes emerged on the factors influencing sub-PrEP adherence. The emerging themes were interpreted and mapped with the HBM constructs namely: medication related barrier, mobility, limited availability and access to PrEP, non-disclosure of PrEP use status, forgetfulness and daily routine challenges, and inadequate knowledge of PrEP benefits. Moreover, another theme emerged as to the strategies used by participants to enhance PrEP use: copying strategies for optimizing adherence to PrEP as shown in Fig. 3. The last theme emerged was participants’ recommendations on the strategies for optimizing PrEP.

The qualitative themes converged with all the quantitative results with complementarity and expansion. Eight meta-themes were developed: Medical and medication related barriers to PrEP adherence; The role of mobility and travel in influencing poor adherence to PrEP; Limited access and availability of PrEP pills contribute to suboptimal adherence; Lifestyle-related factors as drivers of poor adherence to PrEP; Inadequate PrEP knowledge and low perceived benefits of PrEP are drivers of sub-optimal adherence; Non-disclosure PrEP use as a barrier to adherence; and Low perceived HIV risk. Coping strategies and recommendations on strategies to optimize adherence were not measured quantitatively, thus they expanded the findings. Table 3 summarizes the qualitative and quantitative findings with the convergence output.

#### Medical and Medication Related Barriers to PrEP Adherence

##### Experienced Side Effects of PrEP

Participants stated to have experienced side effects which acted as a barrier to optimal adherence to PrEP. During the month 1 and month 6 of follow up interviews, 4.5% and 5% of women respectively reported to have sub-optimal adherence as they wanted to avoid side-effects. In the similar follow up months, 2.4% and 5% of participants reported to have suboptimal adherence as they felt like the PrEP pills were harmful (Table 3). The IDIs revealed that women experienced dizziness, nausea and discomfort following use of PrEP pills. They stated that, to avoid the discomfort brought up by PrEP pills, they had to use the pills inconsistently. She said:

*“I had so much discomfort after using the pills (PrEP), that’s why I was not using them every day. I could take the pills today and skip the next day at least to reduce the side effects, not using them every day as I was told by the healthcare provider”* IDI 23

Moreover, to prevent side effects, some participants had interruptions between the doses as the result of being sick or using other medicines. For example, at month 1, 6 and 12, 6.1%, 12.5% and 10.3% of participants reported to miss PrEP doses as they were sick, and 2.4%, 2.5% and 7.7% skipped PrEP doses as they had other medicines to take respectively (Table 3).

### Medication Characteristics

Participants believed the characteristics of the PrEP pills was a barrier and contributed to sub-optimal adherence. Participants complained of the large pill size stating that the large pill size is difficult to swallow. The requirement that the pills should be taken daily was explained as a burden and imposed laziness in taking them every day. One participant said:

*“Using the pills (PrEP) every day is difficult and burdensome […] also the large size. Sometimes you might feel lazy taking it and skip the doses.”* IDI 25

Participants described that they feel uneasy and anxious when taking the pills. She added:

*“When taking the pills, I sometimes fear, feel uneasy and anxious […]”* IDI 22

### Event-Based PrEP Use or Self-Dosing Shaped by Perceived Risk

Participants stated that using the pills every day is a barrier, thus they only use it as an event-based regimen. Women described that they only use the medication as a situational protection, in an event they are going to meet sexual clients. Further, during the quantitative follow up interviews at months 1, 6 and 12, participants reported 4.3%, 2.5% and 5.1% felt good and 3.1%, 7.5% and 10.3% did not feel at risk of HIV respectively (Table 3), hence perceived no need of using PrEP every day. Also, in the regression analysis, female sex workers who reported never using a condom had a 20% (aPR 1.2, 95% CI 1.02–1.4) relatively increased prevalence of sub-optimal adherence (Table 4). Women stated that, to reduce side effects attributed to the pills, they could not use the pills if they had no plan to have any sexual activity. They further explained that using the pills when just staying home with no sexual encounter is an unnecessary use of medicines. She described:

*“The way I have planned for myself […] I only take the pills when I am going for sex […] to meet sexual clients otherwise I don’t use them at all. Taking the pills while just staying home for what?”* IDI 20

#### The Role of Mobility and Travel in Influencing Poor Adherence to PrEP

Travelling for work and personal issues.

Participants perceived mobility as a barrier to optimal adherence. At baselines, 53.7% of female sex workers reported to have travelled to other cities for sex work in the last 6 months. During the follow up interviews, at months 1, 6 and 12, 29.9%, 62.5% and 69.2% of participants reported being away from home as reason for sub-optimal adherence to PrEP (Table 3). Moreover, in the IDIs, they stated that they travel most of the time for work or personal issues such as attending funerals or wedding ceremonies leaving the pills at home. After further probing, women described, travelling out of the city makes them miss some of the doses and affects their refill schedules when they run out of pills. She said:

*“Sincerely I travel a lot out of the city for work to meet clients, and sometimes clients call us, and pay good if out of the home city […]. My schedule (PrEP refill) found me out of the Tanga city, and I had no pills left. So, I missed those doses”.* IDI 25

#### Limited Access and Availability of PrEP Pills Contributed to Sub-optimal Adherence

##### Distance to Health Facility

Female sex workers described distance to health facilities as a barrier to adherence to PrEP. Leaving far from the health facility affected their access to PrEP service especially when they ran out of pills thus contributing to poor adherence. During the follow up interviews at months 1, 6 and 12, 18.3%, 40.0% and 23.1% of participants stated running out of pills as a reason for sub-optimal adherence respectively (Table 3). In the same follow up period, 14% and 15% reported not having the pills at the time they were supposed to take them respectively. Also, in quantitative interviews, 40.6% of participants reported experiencing financial difficulties due to healthcare spending. In qualitative interviews, participants described that they face travel challenges to get PrEP as they live far from the health facility and that they have no money to pay for transport to and from the health facility. In the regression analysis we found that, women whose only source of income was sex work had a 10% (aPR 1.1, 95% CI 1.02–1.3) increase in prevalence of suboptimal adherence after 12 months of PrEP use (Table 5). Also, at baseline 52.1% of participants reported sex work as the only source of income. On participant said:

*“Some of us live far from here (health facility), paying for transport is difficult […] the pills (PrEP) are not available in nearby health facilities”* IDI 5

##### Unavailability of PrEP in Community Pharmacies

In qualitative interviews, participants explained that unavailability of PrEP services in community pharmacy as a barrier to adherence. They stated that, unavailability of PrEP in community pharmacies makes it hard to access PrEP as they are only available in public health facilities only, which are far from their residence. Women described that, despite the PrEP service being provided free of charge in government facilities, being restricted to those facilities limit access which contribute to suboptimal adherence. One woman described:

*“I have not found these pills in community pharmacies; I once went to ask but I was told it’s not provided […]. They are only given in public health facilities for free”* IDI 5

#### Non-disclosure PrEP Use as a Barrier to Adherence

##### Non-disclosure of PrEP Use Status

Participants described that, not disclosing PrEP use status as a barrier to adherence. It was reported that, not disclosing PrEP use was attributed to the perceived high PrEP stigma (23.5%) and negative reactions they received from family members, partner or friend (21.6%). Participants reported experiencing forced sex as a barrier to disclosure which subsequently affected their PrEP taking behaviour. For example, we found that participants who reported experiencing coercive sex in the past 12 months had a 20% (aPR 1.2, 95% CI 1.1–1.3) increased prevalence of sub-optimal adherence (Table 4). At month12 of follow-up interviews, we found that those who reported experiencing coercive sex in the past 12 months had a 10% (aPR 1.1, 95% CI 1.01- 1.2) increased prevalence of sub-optimal adherence (Table 5). Not wanting others to know if they are using PrEP was reported as a reason for sub-optimal adherence to PrEP during the follow up months 1 (5.5%), month 6 (2.5%) and month 12 (2.6%) (Table 3). Probing further in a qualitative interview, they reported that they did not want even other healthcare workers apart from those providing them PrEP services to know their PrEP use status. They stated to be afraid of discussing with medical doctors if they use PrEP alongside other prescribed medicines. She said:

*“I have not told the doctor if I was using other medicines (PrEP). I was afraid of saying that I am mixing their medicines with PrEP”* IDI 20

#### Lifestyle-Related Factors as Drivers of Poor Adherence to PrEP

##### Forgetfulness Related to Busy Schedules

Women explained that forgetting to take PrEP pills as the barrier to adherence. In quantitative interviews 11.6% (month 1), 37.5% (month 6) and 58.7% (month 12) of participants reported forgetting to take the pills as prescribed due to busy schedules. Also, we found that 20.7% (month 1), 30% (month 6) and 43.6% (month 12) of participants reported simply forgetting to take the pills as the reason for sub-optimal adherence. Additionally, at months 1 and 6 of follow-up interviews, 4.9% and 2.5% of participants reported they could forget to take the pills and sleep through the night respectively (Table 3). Similarly, probing in qualitative interviews, participants stated to forget taking the pills due to busy schedules and pressure of life. **S**he said:

*“I Sometimes forget […] I don’t take them (PrEP), it might take even up to two days when I finally remember that I did not take the pills”* IDI 23

They described that, as humans, they tend to forget especially during busy days, they can forget the pills in the purse or handbag when changing outfits. She added:

*“You know, we are also human, you might forget, or you go somewhere and forget to take it […] during movements of a busy day you switch handbags […] so that’s how it happens.”* IDI 24

##### Forgetfulness Related to Unexpected Events

Experiencing unexpected events was voiced as a barrier to consistent PrEP use. We found that 4.9% (month 1) and 10% (month 6) of participants tend to have change in daily routines as a reason for sub-optimal adherence to PrEP (Table 3). Participants expressed that some circumstances happen during their normal days that prompt them to forget taking the pills. For instance, being invited and attending an unplanned party, binge drinking and khat sessions. Participants described receiving unexpected invitations from their sexual clients thus forgetting to take the PrEP pills. She explained:

*“Sometimes it happens that someone calls you suddenly and you forget to take them (pills) […] I leave abruptly, and when I arrive, I remember I haven’t taken my medication (PrEP).”* IDI 25

At baseline, 73.5% of participants were categorized as being alcohol dependent using AUDIT score, whereas 19.2% reported to use illicit drugs including khat. In quantitative interviews, 12.5% (month 6) and 13.5% (month 12) of participants reported being drunk as the reason for poor adherence to PrEP (Table 3). Participants explained that they go to clubs and they can stay overnight drinking alcohol which interrupts their pill taking schedules. She added:

*“[…] when we go out, we all end up drinking. So, we go to places with music, chew those leaves (khat), we drink, maybe you meet someone like that (sexual client), you sleep over. So honestly, staying completely sober like that no! At first it feels embarrassing, but if you find me already drunk, I don’t feel embarrassed. And I feel like this drinking tendency affects my schedule for taking the pills (PrEP) […]”*. IDI 24

#### Inadequate PrEP Knowledge and Low Perceived Benefits of PrEP are Drivers of Sub-optimal Adherence

##### Limited Understanding of Perceived Benefit of PrEP

Inadequate knowledge and low perceived benefit of PrEP influenced sub-optimal adherence to PrEP. At baseline, 60.6% of participants demonstrated low PrEP knowledge. During the quantitative follow up interviews; 1.8% (month 1), 10.0% (month 2) and 25.6% (month 12) of female sex workers did not find PrEP important as a reason for sub-optimal adherence (Table 3). In qualitative interviews, participants voiced that using PrEP is beneficial although does not need to be used consistently. They stated that not using PrEP consistently had no harm. Moreover, participants described that PrEP prevents all sexually transmitted diseases including syphilis, gonorrhoea and HIV. Some participants were unable to comprehend and articulate the benefits of using PrEP. She said:

*“There are benefits when you use it and thank God that it has protected us […]. It (PrEP) protects us from HIV and other sexually transmitted diseases like HIV, syphilis and gonorrhoea.”* IDI 16

#### Coping Strategies for Optimizing Adherence to PrEP

##### Self-Imposed Schedule

To optimize adherence to PrEP, participants are described to have established their own schedules that help them to keep track of the timing of pill uptake. They stated that they usually take the PrEP pills at a fixed time-either at a specified time in the morning, evening or nighttime before sleeping or going out for work. One woman said:

“[…] because I used to take it in the morning before leaving home for work […]. When I decide to take it, I take it in the morning. I don’t take it when I’m going out. So, if I know, for example, that on the weekend I might go out, then I’ll just take it in the morning.”

##### Visual Cues and Reminders

Participants reported to use visual cues and reminders to make them keep track of the dosing schedules as prescribed. Participants said that they put the PrEP pill container at a place where they can easily see, so that every time they see it, they remember to take it if they have not done so, especially when preparing to leave home for work. Some women describe putting the container on a window or dressing table or in a purse/handbag that they normally use. She said:

*“Sometimes I put them on the makeup table or in my purse… when I see them I […], I remember that I haven’t taken my medicine (PrEP).”* IDI 25

Others said that they use the presence of friends or family as a reminder to take the pills. They explained that their family reminds them to use the pills. Women said that they sometimes tend to take the left-over PrEP pills from friends who stop using them. Also, together with their friends they tend to remind themselves of taking the pills. In the regression analysis, we found that at month 6 of follow up interview, female sex workers living with family and those living with friends had a 10% (aPR 0.9, 95%CI 0.8–0.95) and a 30% (aPR 0.7, 95% CI 0.5–0.9) reduction in the prevalence of suboptimal adherence compared to those living alone, respectively (Table 4). Also, at the 12-month follow-up, female sex workers who lived with family and those who lived with friends had a 10% (aPR 0.9, 95% CI 0.8–0.96) and 20% (aPR 0.8, 95% CI 0.6–0.9) reduced prevalence of suboptimal adherence compared to those who lived alone (Table 5). She added:

*“I keep it (PrEP bottle) on my window […] My friends and I remind each other, and my sister also reminds me since she takes these medicines (PrEP) too. […] My mother asks me if I have taken them (PrEP). This helps a lot, especially because sometimes I forget.”* IDI 5

##### Repackaging of PrEP Pills

Female sex workers described that, to avoid running out of pills when travelling from the city or having a long stay away from home, they tend to repackage the PrEP pills to other packaging materials that are convenient to carry without being noticed. They tend to wrap a few pills equivalent to the length of stay with a piece of paper from the original container. She stated:

*“I travel with my small container […], or if it’s a trip of several days, I just take a few pills, put them somewhere else, and carry them with me. I wrap about fifteen pills in a piece of paper, put them in my purse, and go. I feel at ease.”* IDI 20

#### Proposed Strategies to Improve PrEP Adherence

**Fig. 4:**
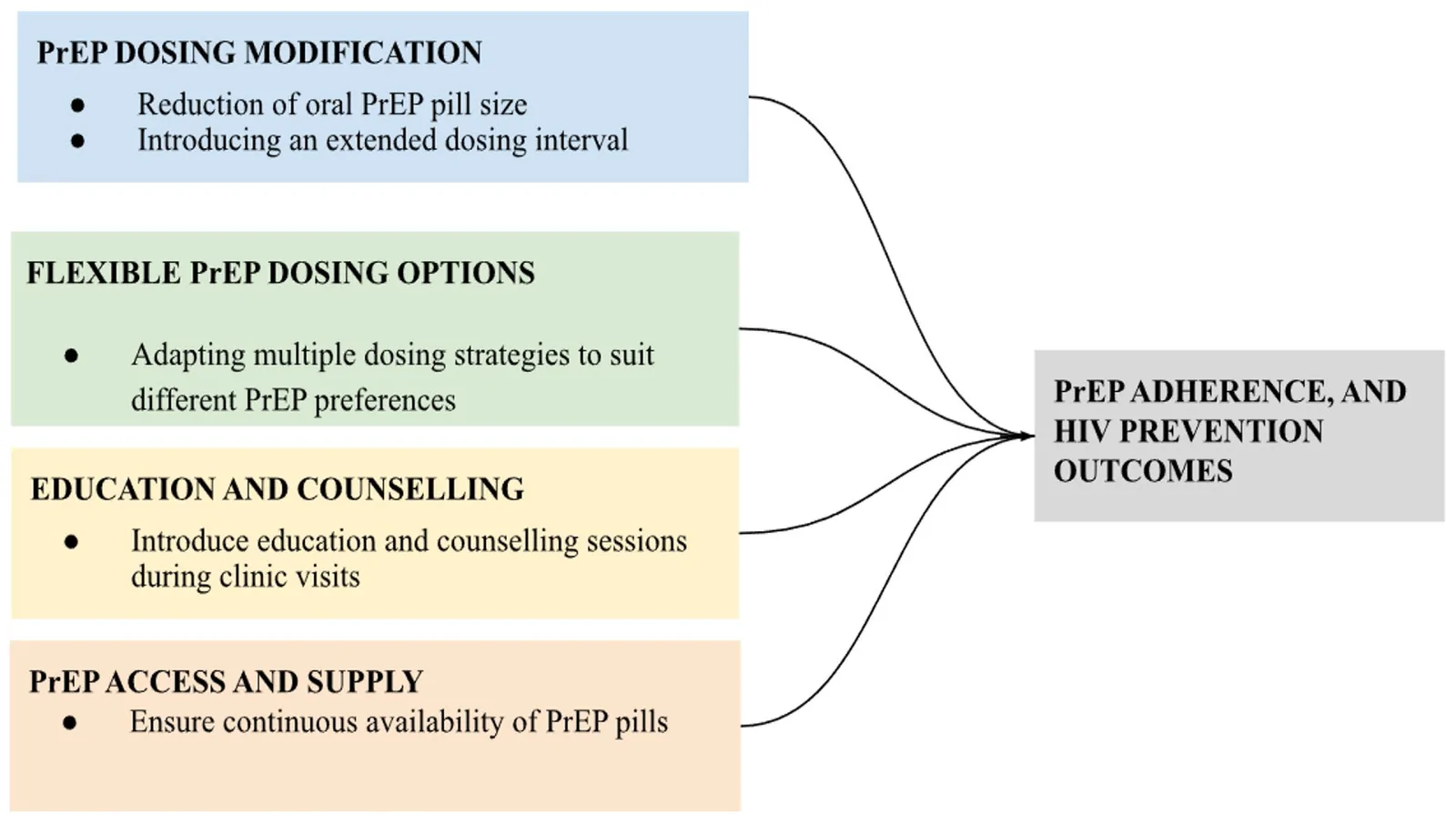
Female sex workers’ recommendations on the strategies to improve PrEP adherence

Four sub-themes emerged regarding strategies to improve PrEP adherence among female sex workers: PrEP dosing modification, introducing multiple dosing options at the PrEP care clinic, enhancing education and counselling sessions, and ensuring continuous availability of PrEP pills (Fig. 4).

### PrEP Dosing Modification

#### Reduction of Oral PrEP Pill Size

One of the beliefs that was communicated by study participants was that reducing the size of the pill would improve PrEP adherence. They highlighted that some female sex workers are afraid of and dislike the current large size of the PrEP pills; therefore, female sex workers are more likely to adhere to and even increase their retention in PrEP services if the pill size is reduced. Some offered examples of the preferred pill size, stating that it should be at least half its current size or exactly the size of piriton^®^ (chlorphenamine). One participant stated:

*“I propose they should reduce the size; the pill is so large. It should be like piriton (antiallergy pills), easy to swallow. In its current form, it’s very large. If a person sees it, they are frightened, it’s this large! They should make it smaller for easy swallowing.”* IDI 20

#### Introducing an Extended Dosing Interval

One of the beliefs that was communicated by some female sex workers was that modification of the dosing interval could likely improve adherence. Participants mentioned that daily dosing is exhausting and very hard to sustain over a long period. They expressed a preference for oral medication, but with an adjustment to the dosing schedule. Women suggested the medication be taken once a week or monthly to lessen the pill burden. She said:

*“I propose they should improve these pills such that, so that when you take them today, you can stay for a week or a month and take them again. This way will prevent tiredness and burdensome results from daily dosing and will improve adherence.”* IDI 25

Some suggested a dose that should be taken only when needed or desired instead of being used daily. One woman explained:

*“They should find us pills that require you to only take once when needed and not daily; daily dosing is a big no. There are not many people who like daily dosing, to be frank.”* IDI 5

### Flexible PrEP Dosing Options

#### Adapting Multiple Dosing Strategies to Suit Different PrEP Preferences

Some female sex workers were of the opinion that introducing multiple PrEP dosing options would improve adherence and retention in care, as most would be attracted to visit health facilities and choose what suits them best. They explained that long-term injections would be most effective because they wouldn’t interfere with their lifestyles, such as drinking alcohol and travelling, and would eliminate concerns about forgetting daily pills due to busy schedules or experiencing side effects from daily dosing.

*“[…] I prefer there be injections for prevention; you only receive one shot every two or three months. Why don’t they bring that one instead of coming here every month? Taking pills daily is very tricky and tiresome, and even adhering is difficult; others are overwhelmed by drunkenness; a long-acting injection can even prevent HIV in those who drink alcohol.”* IDI 4

Participants expressed mixed opinions on the reasons for the preferred modalities. They insisted that different PrEP modalities should be available at the clinic, similar to contraceptives, so that everyone can choose what they prefer. For instance, some female sex workers expressed a fear of injections and a preference for oral dosing, while others favoured long-term injections and were afraid of oral pills. Among participants, some expressed dislike for long-term injections and daily dosing but favoured extended oral dosing, on-demand dosing or periodic dosing. One participant stated:

*“I am afraid of injections, and I know there are others who feel the same, while some people prefer injections and others prefer pills. Personally, I prefer pills, but not daily. There should be options that allow each person to choose their preferred method, whether injections or pills. Ideally, this could work so that when visiting the clinic, an individual can indicate their preference for a long-acting injection or pills or other modalities as available. These options should be readily available and provided free of charge.”* IDI 5

#### Enhancing Education and Counselling Sessions

Participants explained that the Ministry of Health should emphasize education and counselling sessions. They demanded that, if possible, dedicated sessions should be established to provide education and counselling on HIV prevention and proper use of PrEP medications among female sex workers and other groups at high risk of HIV. They indicated that continuity in providing these sessions would likely improve adherence to PrEP.:

*“I recommend continuing to emphasize education so people become more informed about their HIV risks and use medication properly.”* IDI 26

Participants mentioned that, even with various HIV prevention methods available, effective counselling needs to be improved because people tend to forget or overlook important information over time. A woman said:

*“It is important to provide counselling periodically to individuals taking these medications, as we tend to forget sometimes. Counselling ensures that users understand the purpose of the medication and are guided on proper use […].”* IDI 17

### PrEP Access and Supply

#### Ensure Continuous Availability of PrEP Pills

Female sex workers were certain that ensuring the continuous availability of PrEP pills would improve adherence. They voiced that availability and access of the pills are essential for prevention, as one could not adhere due to frequent stockouts of pills at the health facilities. They emphasized that, because of increasing risky sexual behaviour, adhering to the pills is crucial to protect themselves from HIV acquisition.

*“The government should ensure these pills are readily available and accessible to all, as they are highly effective in preventing HIV. Risky sexual encounters with our clients are increasing, often taking place in secluded areas such as parks, cemeteries, or narrow alleys in local neighbourhoods, making access to preventive medication essential. Ensuring the consistent availability of these pills will prevent stockouts and allow us to take them daily without interruption.”* IDI 9

## Discussion

We determined the prevalence, factors, and reasons for suboptimal adherence among female sex workers in the city of Tanga, Tanzania. We also explored women’s own recommendations for strategies to improve adherence to daily oral PrEP among female sex workers. The prevalence of suboptimal adherence increased from 85.6% at 6 months to 90.4% at 12 months. Living with family or friends was linked to a lower prevalence of suboptimal adherence. Women whose only income source was sex work, who experienced coercive sex, or who did not use a condom were more likely to have suboptimal adherence. Common reasons for suboptimal adherence included being away from home, running out of pills, forgetting to take pills, being busy, not perceiving PrEP as beneficial, perceiving no HIV risk, being sick, or being drunk. Female sex workers suggested that modifying PrEP dosing, introducing multiple dosing options at the PrEP care clinic, enhancing education and counselling sessions, and ensuring continuous availability of PrEP pills would improve adherence.

Most female sex workers in our study had sub-optimal adherence to daily oral PrEP. The prevalence of suboptimal adherence was higher than in a cross-sectional study conducted in Northern Tanzania, which reported a high prevalence (56.7%) among female sex workers [22]. The differences observed could be due to variations in study design; our study was nested in a quasi-experimental trial with adherence measured at months 6 and 12, whereas their study measured adherence cross-sectionally. Additionally, differences in adherence measures may have contributed to the variation in prevalence. We also noted a trend of increasing suboptimal adherence from months 1 through 12, similar to a study in Benin [8], which reported a 31% decline in adherence every six months. A decline in optimal adherence among women was also seen in demonstration and open trials from sub-Saharan Africa, India, and the USA, showing a drop from 80 to 13% within 16 weeks [19], highlighting the difficulty in maintaining initial enthusiasm for a new prevention strategy over time [8]. It is important to recognize that women often use PrEP only during periods they perceive as high-risk for HIV [2, 23]. The rise in suboptimal adherence in our study could be partly due to unmet needs among female sex workers in Tanzania, including stigma, discrimination, and access [24] issues. Notably, a large majority (88%) of female sex workers in Tanzania prefer long-acting injectable PrEP over daily oral PrEP [25]. Therefore, efforts should focus on interventions to enhance and sustain adherence among PrEP users by involving pharmacists in adherence counselling [26].

The high prevalence of suboptimal adherence to daily PrEP among female sex workers was associated with not using condoms with clients. These findings are similar to those of the Ugandan study, which reported that female sex workers who did not use condoms with clients had reduced odds of optimal adherence to daily oral PrEP [27]. Increased likelihood of poor adherence among those not using a condom could be due to a false perception of low HIV risk. This false perception of HIV risk and the need for money could have led participants to not adhere to PrEP, for example, engaging in condomless sex for higher payment, since it’s the only main income source despite not taking PrEP pills as recommended. According to the health belief model [28], individuals tend to use an intervention if they perceive themselves to be at high risk of infection; thus, a false perception of low risk could prevent them from using PrEP as recommended among non-condom users.

Moreover, experiencing any form of gender-based violence, especially from a partner, such as coercive sex, is linked to poor adherence to PrEP [29]. In our study, more than a quarter of participants experienced coercive sex, which was significantly associated with low adherence to oral PrEP. It is important to note that client-perpetrated violence is associated with depression and anxiety, which can further compromise optimal PrEP use among female sex workers [30]. Therefore, interventions to address genderbased violence should be integrated with PrEP services, especially for female sex workers who face increased risk of both sexual violence and HIV infection [31].

The findings on the reasons for sub-optimal adherence are consistent with those of other studies worldwide. For example, a systematic review of HIV pre-exposure prophylaxis uptake, retention, and adherence among female sex workers in sub-Saharan Africa identified being busy, running out of pills, perceiving no risk of HIV, and forgetfulness as common reasons for sub-optimal adherence [32]. Reviews of adherence studies among female sex workers globally reported mobility, hospitalisation, and substance use, such as alcohol, as reasons for sub-optimal adherence, similar to our findings [33, 34]. An early (2016–2017) systematic review of adherence to oral PrEP from prospective randomised controlled trials and implementation studies found similar factors, such as perceptions of low benefits or efficacy in protecting against HIV, busy schedules, and false perceptions of low HIV risk, as reasons for sub-optimal adherence to daily oral PrEP [10]. Therefore, a complex interplay of factors contributes to sub-optimal adherence to daily oral PrEP, highlighting the need for a client-centred approach to improve adherence.

Nevertheless, we noted that female sex workers living with families or friends had a lower chance of suboptimal adherence compared to those living alone. Similar findings were reported in Tanzania, where female sex workers living with family had increased odds of good adherence [23]. Also, a qualitative study of adolescents and young women in South Africa described supportive families and partners as a critical component in improving adherence [35]. Supportive family and friends are fundamental in motivating women to adhere to PrEP by reminding them to take the pills on time [35, 36]. These findings highlight the need to raise awareness among community members, especially at the family level, which can positively change the behaviour of unsupportive family members.

Studies are ongoing to develop PrEP formulations that mirror contraceptive methods to optimize adherence and reduce HIV incidence [4]. In our study, female sex workers recommended reducing pill size and/or extending the dosing interval to lessen the burden and make pill taking easier. This strategy aligns with studies advocating for extended dosing options for PrEP. For instance, a study on islatravir paves the way for long-acting oral PrEP, which enables monthly dosing due to its long intracellular half-life of 78.5–128 hours [37, 38].

The World Health Organization recommends introducing multiple PrEP dosing options as an intervention to improve adherence [14]. The availability of various PrEP modalities offers choices that cater to individual needs and preferences. These modalities include, but are not limited to, daily dosing, on-demand, vaginal dapivirine ring, and long-acting injectable PrEP (cabotegravir and lenacapavir) [39]. Our study aligns with these recommendations, as participants believed that offering multiple PrEP options would better suit individual needs and preferences. It is important to note that female sex workers have different preferences for dosing modalities [25, 39]. A study conducted in Tanzania reported high acceptability of various dosing modalities and advocated for their inclusion in prevention services [25]. Therefore, our findings support the WHO recommendations and previous studies’ suggestions for introducing multiple PrEP modalities in Tanzania. Moreover, strategies to improve coping skills should be investigated as our study demonstrated that, women had coping skills as part of the adaptive mechanisms to overcome barriers to sustained PrEP use. These coping skills acted as triggers to remind them on using the pills.

It has been demonstrated that a single strategy is not effective in optimizing adherence [13]. Therefore, ensuring a constant supply and/or access to PrEP options should be combined with other strategies such as tailored adherence counselling or education programs. To date, in Tanzania, only daily oral PrEP is available for use as PrEP despite the WHO recommendation of long-acting injectable PrEP and the dapivirine vaginal ring. It’s high time for Tanzania to adopt a multiple PrEP modalities model into the HIV prevention programme by incorporating long-acting injectable PrEP (lenacapavir and cabotegravir) in addition to daily oral PrEP to improve adherence.

### Strengths and Limitations

The adherence measures used in this study are subject to social desirability and recall bias, which could affect the validity of the findings. However, we used different adherence measurements, and all measures correlated well, indicating that the findings on suboptimal adherence reflected the actual adherence pattern. It is recommended to use more than one adherence measure to increase the validity of the findings, as one method can address the weaknesses of another [40]. Notably, there was a loss to follow-up of study participants, i.e., only 52.4%, 62.3% and 60.1% returned for interviews at months 1, 6, and 12, respectively. We compared the baseline characteristics of all participants who returned to the scheduled interviews, and we observed that the loss to follow-up was not selective, reflecting comparable characteristics with those who did not return. The measured adherence did not align with the periods of increased HIV risk as female sex workers could have intentionally missed PrEP pills due to the reduced perceived. However, our previous paper noted a high prevalence (~ 90% at months 6 and 12) of suboptimal adherence during increased HIV risk among the cohort of female sex workers [2]. Nonetheless, in our study, we did not determine HIV incidences in relation to the adherence pattern, and adherence was measured at a single point during scheduled visits instead of continuously, which may overestimate the level of suboptimal adherence. Additionally, data collected only from female sex workers should not be generalized to other members of key populations.

## Conclusion and Recommendation

The prevalence of suboptimal adherence to oral PrEP among female sex workers increases consistently from month 1 through 12. Female sex workers living with family or friends are less likely to have suboptimal adherence to PrEP. Suboptimal adherence is linked to medical and medication related barriers, mobility and travel, limited access and availability of PrEP pills, lifestyle-related factors, inadequate PrEP knowledge and low perceived benefits of PrEP, non-disclosure PrEP use to family, friends and healthcare workers, and low perceived HIV risk. Female sex workers have coping strategies that enable them to adhere to daily oral PrEP. Moreover, female sex workers suggest that modifying PrEP dosing, introducing multiple PrEP options, providing education and counselling, and ensuring access and availability could improve adherence. The study highlights the influence of various factors on PrEP adherence and underscores the need for implementation research on different dosing strategies, along with better availability, accessible services, and tailored counselling programs.

## Figures and Tables

**Table 1:** Distribution of participants’ sociodemographic characteristics by follow-up duration among female sex workers in Tanga, Tanzania

Variables	Month 0 N = 313	Month 1 N = 164	Month 6 N = 195	Month 12 N = 188
Mean age (SD)	27.7 (6.2)	28.4 (6.1)	28.1 (6.0)	28.4 (6.1)
Education level				
No formal education	39 (12.5)	4 (2.4)	19 (9.8)	20 (10.7)
Primary	130 (41.7)	87 (53.0)	90 (46.4)	85 (45.5)
Secondary +	143 (45.8)	73 (44.5)	85 (43.8)	82 (43.9)
Ever married	91 (29.2)	55 (33.5)	57 (29.4)	58 (31.0)
Having children	272 (86.9)	112 (80.0)	165 (85.1)	166 (88.8)
Living arrangements				
Alone	37 (11.9)	19 (11.6)	19 (9.8)	27 (14.4)
Family	237 (76.0)	117 (71.3)	154 (79.4)	134 (71.7)
Friends/Others	38 (12.2)	28 (17.1)	21 (10.8)	26 (13.9)
Median (IQR) number of people currently living with study participants	3 (2–5)	3 (2–5)	3 (2–5)	3 (2–5)
Sex work is the only source of income	163 (52.1)	100 (61.0)	102 (52.3)	91 (48.7)
Perceived HIV risk				
Medium/High risk	254 (81.4)	136 (82.9)	158 (81.4)	156 (83.4)
Low/No risk	49 (15.7)	24 (14.6)	31 (16.0)	26 (13.9)
Don’t know the risk	9 (2.9)	4 (2.4)	5 (2.6)	5 (2.7)
Low PrEP knowledge	189 (60.6)	103 (62.8)	123 (63.4)	115 (61.5)
High PrEP stigma	72 (23.5)	36 (22.5)	43 (22.5)	48 (26.2)
High PrEP self-efficacy	310 (99.4)	163 (99.4)	192 (99.5)	186 (99.4)
Number of sexual clients last month				
< 10	69 (22.2)	37 (22.6)	51 (26.3)	48 (25.7)
10–29	107 (34.4)	55 (33.5)	58 (29.9)	63 (33.7)
30 +	135 (43.4)	72 (43.9)	85 (43.8)	76 (40.6)
Adequate social support	154 (51.9)	81 (51.9)	98 (53.3)	98 (55.1)
Arrested by police in the last 12 months	80 (25.6)	42 (25.6)	52 (26.8)	46 (24.6)
Coercive sex in the last 12 months	93 (29.8)	49 (29.9)	55 (28.4)	57 (30.5)
Mobility for sex work in the last 6 months	167 (53.7)	94 (57.3)	102 (52.6)	102 (54.5)
Frequency of condom use with clients				
Every time	69 (22.3)	27 (16.7)	39 (20.2)	34 (18.3)
Several times	107 (34.5)	65 (40.1)	78 (40.4)	72 (38.7)
Few times	113 (36.5)	55 (34.0)	60 (31.1)	62 (33.3)
Never used	10 (3.2)	6 (3.7)	6 (3.1)	10 (5.4)
No response	11 (3.6)	9 (5.6)	10 (5.2)	8 (4.3)
Lubricant use during sex	157 (77.7)	90 (75.6)	105 (79.6)	100 (79.4)

IQR- Interquartile range; PrEP-Pre-Exposure Prophylaxis of HIV

Some percentages do not sum to exactly 100% due to rounding off

**Table 2:** In-depth interview participants’ characteristics

IDI number	Age	Education level	PrEP use status
1	36	Primary	Suboptimal adherence
2	35	Primary	Suboptimal adherence
3	31	Secondary	Suboptimal adherence
4	42	Secondary	Suboptimal adherence
5	30	Primary	Suboptimal adherence
6	30	Tertiary	Suboptimal adherence
7	28	Primary	Suboptimal adherence
8	38	Primary	Suboptimal adherence
9	32	Primary	Suboptimal adherence
10	34	Secondary	Suboptimal adherence
11	23	Secondary	Suboptimal adherence
12	32	Tertiary	Suboptimal adherence
13	32	Primary	Suboptimal adherence
14	25	Secondary	Adherence
15	27	Primary	Adherence
16	23	Secondary	Discontinue
17	26	Primary	Discontinue
18	47	Primary	Discontinue
19	23	Primary	Discontinue
20	36	Primary	Discontinue
21	20	Secondary	Discontinue
22	24	Primary	Discontinue
23	28	Secondary	Discontinue
24	27	Secondary	Discontinue
25	32	Secondary	Adherence
26	34	Secondary	Suboptimal adherence

**Table 3:** Quantitative insights and qualitative findings with convergence outcomes

Quantitative insights				Qualitative findings	Convergence outcomes: Meta-theme
Self-reported insights on adherence	Month 1 (n=164) (%)	Month 6 (n = 40) (%)	Month 12 (n = 39) (%)		
Away from home	29.9	62.5	69.2	Mobility	Convergence: The role of mobility and travel in influencing poor adherence to PrEP
Ran out of pills	18.3	40.0	23.1	Limited access and availability of PrEP	Convergence and expansion: Limited access and availability of PrEP pills contributed to sub-optimal adherence
Did not have the pills at the time they were supposed to be taken	14	15	0		
Busy with other things	11.6	37.5	58.7	Forgetfulness and daily Routine Challenges	Convergence: Lifestyle-related factors as drivers of poor adherence to PrEP
Simply forgot	20.7	30	43.6		
Had a change in daily routine	4.9	10	0		
Fell asleep/slept through the time	4.9	2.5	0		
Were drunk	0	12.5	13.5		
Wanted to avoid side effects	4.9	5	0	Medication related barriers	Convergence: Medical and medication related barriers to PrEP adherence
Felt like the drug was toxic/harmful	2.4	5	0		
Had problems taking the pill at the specified time	4.9	5	0		
Felt sick or ill	6.1	12.5	10.3		
Having other medicines to take	2.4	2.5	7.7		
Did not find it important	1.8	10	25.6	Inadequate knowledge on the benefits of PrEP	Convergence and expansion: Inadequate PrEP knowledge and low perceived benefits of PrEP are drivers of sub-optimal adherence
Did not want others to notice you taking medication	5.5	2.5	2.6	Non-disclosure PrEP use status	Convergence: Non-disclosure PrEP use as a barrier to adherence
Felt good	4.3	2.5	5.1	Low perceived risk of HIV	Convergence: Low perceived HIV risk undermines adherence to PrEP
Did not feel at risk of HIV	3.1	7.5	10.3		
Not measured				Expansion: Coping strategies	
Not measured				Expansion: Recommendations on strategies	

**Table 4:** Modified Poisson regression analysis on the factors associated with suboptimal PrEP adherence (at month 6) among female sex workers

Variables	cPR	95%CI	p-value	aPR	95%CI	p-value
Education level						
No formal education	ref			ref		
Primary	1.2	0.9–1.6	0.3	1.2	0.9–1.6	0.24
Secondary +	1.2	0.9–1.7	0.16	1.2	0.9–1.6	0.29
Marital status						
Never married	ref			ref		
Married or previously married	0.9	0.8–1.04	0.14	0.9	0.8–1.01	0.12
Living arrangements						
Alone	ref			ref		
Family	0.9	0.8–0.9	< 0.001	0.9	*0.8–0.95*	*0.002*
Friends	0.7	0.5–0.9	0.01	0.7	*0.5–0.9*	*0.01*
Perceived HIV risk						
High risk	ref			ref		
Low/No risk	0.9	0.7–1.1	0.21	0.95	0.8–1.2	0.59
Do not know the risk	0.7	0.3–1.4	0.30	0.7	0.4–1.3	0.23
PrEP knowledge						
High	ref			ref		
Low	0.9	0.8–1	0.05	0.96	0.9–1.1	0.43
Number of sexual clients last month						
< 10	ref			ref		
10–29	0.99	0.8–1.2	0.89	0.97	0.8–1.1	0.68
≥ 30	1.1	0.95–1.3	0.20	1.1	0.9–1.2	0.33
Coercive sex in the last 12 months						
No	ref			ref		
Yes	1.1	1.01–1.3	0.03	1.2	*1.1–1.3*	*0.004*
Frequency of condom use with clients						
Every time	ref					
Several times	1.1	0.9–1.3	0.38	1.1	0.9–1.3	0.35
Few times	1.1	0.9–1.3	0.55	1.1	0.9–1.3	0.47
Never used	1.2	1.1–1.4	0.01	*1.2*	*1.1–1.5*	*0.01*
No response	0.8	0.6–1.3	0.47	0.9	0.6–1.4	0.71

aPR: adjusted prevalence ratio; cPR: crude prevalence ratio; PrEP- HIV Pre-exposure Prophylaxis; ref: reference

**Table 5:** Modified Poisson regression analysis on the factors associated with suboptimal PrEP adherence (at month 12) among female sex workers

Variable	cPR	95% CI	p-value	aPR	95%CI	p-value
Marital status						
Never married	ref					
Married or previously married	0.9	0.8–1.02	0.12	0.9	0.8–1.01	0.06
Living arrangements						
Alone	Ref					
Family	0.9	0.8–0.96	< 0.001	*0.9*	*0.8–0.96*	*0.003*
Friends	0.8	0.7–0.98	0.03	*0.8*	*0.6–0.9*	*0.01*
Sex work is the only source of income						
No	ref					
Yes	1.1	0.97–1.2	0.17	*1.1*	*1.02–1.3*	*0.02*
PrEP knowledge						
Low	Ref					
High	1.1	1.01–1.2	0.03	1.1	0.99–1.2	0.11
Social support						
Adequate	ref					
Inadequate	0.9	0.8–1.03	0.16	0.9	0.8–1.01	0.07
Coercive sex in the last 12 months						
No	ref					
Yes	1.1	0.98–1.2	0.12	*1.1*	*1.01–1.2*	*0.04*
Frequency of condom use with clients						
Every time	ref					
Several times	1.04	0.9–1.2	0.60	1.03	0.9–1.2	0.70
Few times	1.04	0.9–1.2	0.58	1.01	0.9–1.2	0.90
Never used	1.1	1–1.3	0.05	*1.2*	*1.02–1.4*	*0.03*
No response	0.8	0.6–1.3	0.45	0.9	0.6–1.3	0.57

aPR: adjusted prevalence ratio; cPR: crude prevalence ratio; PrEP- HIV Pre-exposure Prophylaxis; ref: reference

## Data Availability

All data are available upon reasonable request. The request for data can be sent to the PREPTA principal investigator: Prof. Elia J Mmbaga; elia.mmbaga@medisin.uio.no.

## Abbreviations

HIV: Human immunodeficiency virus
PrEP: Pre-Exposure prophylaxis
PREPTA: Pragmatic trial for HIV pre-exposure prophylaxis roll-out in Tanzania
